# Lung cancer screening eligibility and recruitment during routine care by pulmonologists: barriers and new opportunities in the Brazilian public healthcare system

**DOI:** 10.36416/1806-3756/e20240071

**Published:** 2024-07-29

**Authors:** Fábio Munhoz Svartman, Marina Ilha de Azambuja, Eduarda de Albuquerque Palma, Ana Paula Garcia Sartori, Maurício Mello Roux Leite

**Affiliations:** 1. Hospital Nossa Senhora da Conceição, Porto Alegre (RS) Brasil.; 2. Hospital de Clínicas de Porto Alegre, Porto Alegre (RS) Brasil.; 3. Hospital Ernesto Dornelles, Porto Alegre (RS) Brasil.

## TO THE EDITOR:

Lung cancer screening (LCS) with low-dose CT (LDCT) reduces mortality in high-risk patients and its practice is now widely recommended.^1^ ^-^ ^3^ However, large-scale implementation and adherence to screening remain low,^4^ and difficulties in screening implementation may be greater in low- and middle-income countries such as Brazil.^3^

Organized, population-based programs are the preferable approach for cancer screening, due to their greater scope and impact.^5^ However, initiatives with features of opportunistic screening (i.e., offering screening during a medical visit for another reason) have demonstrated positive outcomes across diverse real-world scenarios.^6^ ^,^ ^7^

Our group performs LCS with LDCT in patients with chronic, stable lung diseases (mostly COPD) who are regularly followed by pulmonologists in a Brazilian public hospital.^8^ Data from the program implementation demonstrated adequate rates of early-stage diagnoses and treatments with curative intent.^8^ However, little is known about the effective recruitment potential of LCS programs for patients with respiratory diseases. The present study aimed to identify the number of patients who are truly eligible for screening in this context, as well as physicians’ adherence to recruiting eligible patients. Secondarily, we aimed to identify factors that hinder a greater volume of LCS program recruitment.

This was a cross-sectional study involving all patients seen in November of 2022 at the institution’s pulmonology outpatient clinic. Demographic data and presence of the screening inclusion criteria in accordance with the institutional program were retrospectively reviewed. Inclusion criteria for our LCS program are: 1) being between 55 and 80 years of age; 2) being a current smoker or a former smoker for less than 15 years; and 3) having a smoking history of 30 pack-years or more. Reasons for not requesting LDCT were actively sought by reviewing the data recorded by the attending physicians, and exam requests were searched in the hospital’s radiology system. The month of November of 2022 was used for index consultations, the following period of one year being evaluated to detect LDCT screening. This was a convenience sample, and all individual outpatient appointments in the specified month were analyzed. The program’s exclusion criteria are: current suspicion of cancer, personal history of lung cancer, and presence of comorbidities that, in the judgment of the attending physician, limit the patient’s life expectancy and/or clinical performance for curative treatment. Descriptive statistics were used for main results, and the chi-square and the Fisher’s exact tests were used for exploratory comparisons between the group of patients effectively screened and those in which screening was not performed despite confirmed eligibility. This study is the initial stage of a project that proposes evaluating future educational interventions to increase enrollment in screening. The project was approved by the institution in its ethical and methodological aspects (CAAE no. 77187623.1.0000.5530).

**Figure 1:**
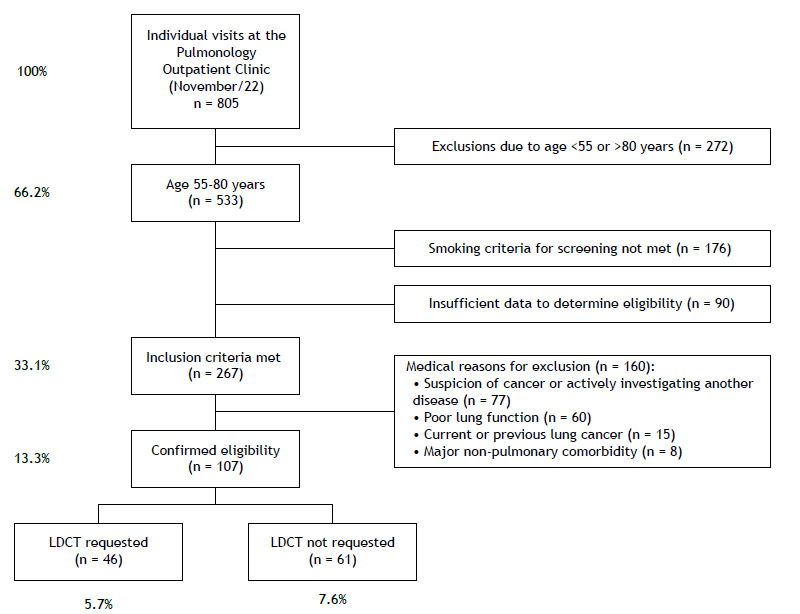
Flow chart of the patient selection process. Criteria used by the program: current smoking or smoking cessation less than 15 years ago; and smoking history of 30 pack-years or more.

Figure 1 presents the flowchart of evaluated patients and the causes identified for non-enrollment in the program. Of the 805 patients seen during the study period, 532 (66.2%) were in the target age range for screening, and 267 (33.1%) met all inclusion criteria. In 40% of these (n = 107; 13.3% of the total) absence of exclusion criteria could be verified (i.e., eligibility confirmed). The most common medical reasons for exclusion were current suspicion of cancer and/or active investigation of another medical condition and poor lung function. There were insufficient data in the medical records of 90 patients (11% of the total sample) to determine eligibility. Table 1 presents the demographic data of patients who met all inclusion criteria and of the subgroup with confirmed eligibility.

**Table 1:** In A, demographic profile, age range, and confirmed eligibility for lung cancer in the patients who met all inclusion criteria (N = 267). In B, association between demographic variables and low-dose CT performance of patients with confirmed eligibility.^a^

A			B			
Variable	Result		Variable	LDCT	No LDCT	p
				(n = 46)	(n = 61)	
Gender			Gender			
Female	170 (63.7)		Female	28 (60.9)	49 (80.3)	0.027*
Male	97 (36.3)		Male	18 (39.1)	12 (19.7)	
Age, years	66.9 ± 6.5					
Age range, years			Age range, years			
55-60	55 (20.6)		55-60	15 (32.6)	13 (21.3)	0.738**
61-64	43 (16.1)		61-64	9 (19.6)	12 (19.7)	
65-67	54 (20.2)		65-67	8 (17.4)	16 (26.2)	
68-71	46 (17.2)		68-71	6 (130)	11 (18.0)	
72-75	37 (13.9)		72-75	3 (6.5)	3 (4.9)	
76-80	32 (12.0)		76-80	5 (10,9)	6 (9,8)	
Confirmed eligibility						
Yes	107 (40.1)					
No	160 (59.9)					

LDCT: low-dose computed tomography. ^a^Values expressed as n (%) or mean ± SD. *Chi-square test. **Fisher’s exact test.

Of the 107 patients with confirmed eligibility, LDCT was requested and not requested in 46 (43%) and 61 (57%) cases, respectively. The comparison between these two groups revealed a statistically significant difference in the proportion of women, who constituted 60.9% of the screened group and 80.3% of the non-screened group (p = 0.027). In other words, of the 77 women with confirmed eligibility, 28 (36.3%) were screened, in contrast to 18 of the 30 men (60%) in the same situation. There was no difference regarding age between the groups (p = 0.738).

These results point to four main conclusions: 1) at least one third of patients in this context met the inclusion criteria for screening, although only 13% had confirmed eligibility; 2) underreporting of clinical data was significant, pointing to a potential greater number of eligible patients; 3) even in patients with confirmed eligibility, the effective inclusion rate was low (43%); 4) patient characteristics, such as gender, might have an impact on the decision to request LDCT.

This study has several limitations, including its retrospective single-center nature and the small number of participants. However, it was possible to quantify and detail barriers, some of which were inherent to the nature of our program, such as the number of patients with medical contraindications. On the other hand, barriers with the possibility of intervention for improvement include underreporting and, in particular, the low inclusion rate of patients with confirmed eligibility. Our study does not allow us to conclude whether this low inclusion rate was due exclusively to physician nonadherence or to patient factors (such as possible refusals to undergo screening). A finding that deserves further investigation is the lower rate of LDCT requests for women, raising awareness of possible gender inequalities. The small sample size and the absence of analysis of potentially confounding factors limit conclusions about this finding. Unfortunately, ethnicity and socioeconomic data were unavailable, precluding inferences about possible inequities in these aspects as well. Efforts to reduce inequities in LCS have recently led to important advancements,^9^ ^,^ ^10^ and we believe that such programs should have a special focus on systematically monitoring this issue.

In conclusion, the number of patients with chronic respiratory diseases and confirmed eligibility for LCS appears significant, although effective inclusion remains low. We believe that educational interventions and periodic reminders to physicians are necessary in such scenarios. Pulmonologists’ expertise in order to select and conduct screenings carefully in a setting with the necessary resources is crucial for this high-risk subgroup of patients.
